# Separation and Enrichment of Lectin from Zihua Snap-Bean (*Phaseolus vulgaris*) Seeds by PEG 600–Ammonium Sulfate Aqueous Two-Phase System

**DOI:** 10.3390/molecules22101596

**Published:** 2017-09-22

**Authors:** Bin Jiang, Yongqiang Yuan, Xiaoqing Zhang, Zhibiao Feng, Chunhong Liu

**Affiliations:** Department of Applied Chemistry, Northeast Agricultural University, No. 600 Changjiang Road, Xiangfang District, Harbin 150030, China; jiangbin@neau.edu.cn (B.J.); yyqyinghua@yahoo.com (Y.Y.); xqz2015yh@yahoo.com (X.Z.); liuchunhong@neau.edu.cn (C.L.)

**Keywords:** aqueous two-phase system, lectin, separation and enrichment, Zihua snap-bean seeds

## Abstract

A fast and efficient method based on a polyethylene glycol (PEG) 600/(NH_4_)_2_SO_4_ aqueous two-phase system for extracting lectin from Zihua snap-bean (*Phaseolus vulgaris*) seeds was established. According to a Box–Behnken design (BBD), involving four factors at three levels each subjected to analysis of variance (ANOVA) and response surface analysis, the protein recovery and the purification factor of lectin in the top phase were used as the response values of the variance analysis to acquire the multivariate quadratic regression model. SDS–PAGE electrophoresis and the hemagglutination test were used to detect the distribution of lectin in the aqueous two-phase system (ATPS). The obtained data indicated that lectin was preferentially partitioned into the PEG-rich phase, and the ATPS, composed of 15% (NH_4_)_2_SO_4_ (*w*/*w*), 18% PEG 600 (*w*/*w*), 0.4 g/5 g NaCl and 1 mL crude extract, showed good selectivity for lectin when the pH value was 7.5. Under the optimal conditions, most of the lectin was assigned to the top phase in the ATPS, and the hemagglutination activity of the purified lectin in the top phase was 3.08 times that of the crude extract. Consequently, the PEG 600/(NH_4_)_2_SO_4_ aqueous two-phase system was an effective method for separating and enriching lectin directly from the crude extract of Zihua snap-bean seeds.

## 1. Introduction

Due to the advantage of being able to bind specifically to monosaccharides, polysaccharides and glycoproteins [1], plant lectin has played a huge role and value in medicine, materials science and agronomy [2,3]. Lectins are proteins, or glycoproteins, with at least one carbohydrate or derivate binding site without catalytic function (nonenzymatic) or immunological characteristics [1]. Lectins are highly sought-after proteins due to their wide potential in many cellular and molecular recognition processes, as well as in pharmacology, biochemistry, medicine and clinical analysis [4,5].

Lectins are widely distributed in organisms such as in viruses, fungi, bacteria, animals and plants [6]. Plant lectins are classified into seven families according to their evolutionary and structural characteristics [7], and among these families, lectins of the Leguminosae family are, thus far, the most-studied group. At present, nearly a thousand species of plant lectins have been found, among which leguminous plant lectins are the most abundant, accounting for more than 600 species, of which more than 70 species have been isolated and purified [8].

Zihua snap beans (*Phaseolus vulgaris*) are an endemic species which distributes in the northeast areas of China. More than 20% of the dry weight of Zihua snap-bean seeds is protein. These proteins present high nutritional and biological values with the reasonable amino acid composition and relatively high content of lectin.

Being the traditional method for protein separation, the ammonium sulfate precipitation method was also used to extract lectins. The disadvantage of this method was that it is time-consuming and affords low yield [9]. The affinity chromatography method greatly improved the yield of lectin, but it was costly and difficult to be scaled-up industrially [10,11]. Therefore, it is particularly important to establish a quick and cost-effective technique for extracting lectin to increase selectivity and capacity, and reduce overall costs. With regard to downstream processing in protein purification, aqueous two-phase extraction (ATPE) has been increasingly used to separate or concentrate important biomolecules including proteins [12], and using a PEG/ammonium sulfate system in the extraction and separation of protein has huge advantages [13,14].

Currently, liquid–liquid extraction techniques are also attempted for the separation and purification of lectins. One is the reverse micelles extraction technique [15,16], and another is ATPE. Each phase of the aqueous two-phase system (ATPS) contains 80% to 90% water [17], which provides a mild environment to protect the bioactivity of lectin.

In 2010, Nascimento [18,19] studied the distribution of *Canavalia brasiliensis* lectin (ConBr) in a PEG 600/phosphate system and PEG 600/sodium citrate system, and purified the ConBr with purity of 73.04% and 98%, respectively. The results proved that PEG 600 could be a better choice for extracting lectin. Soares [20] reported that Concanavalin A (Con A) was extracted and purified from the crude extract of *Canavalia ensiformis* seeds in a PEG 8000/citrate system, and the obtained purification factor was 11.5. Nascimento [21] extracted lectin from *Cratylia mollis* seeds with a PEG 8000/sodium citrate system, and the purification factor was 13.28. The results showed that a PEG/salt system has great advantages in extracting lectins.

In this study, seeds of the Zihua snap bean, an endemic species in northeast China, were chosen to be the raw materials from which to extract and separate lectin. A PEG 600/ammonium sulfate system was used to extract and separate lectin from Zihua snap-bean seeds. The purpose of the present work was to establish a method for selective separation of lectin directly from the crude extract of Zihua snap-bean seeds, based on an alternative method to fractionate the lectins. Furthermore, the commercial value of Zihua snap beans could be improved if they are a source of lectin.

## 2. Results and Discussion

### 2.1. Single-Factor Variable Analysis

In order to investigate the effect of different variables on the distribution of lectins in ATPS, each parameter—including concentration of ammonium sulfate, concentration of PEG 600, NaCl content and pH—was changed while others were fixed, to form the ATPS. Based on the protein recovery in the top phase (*Y*), the hemagglutinating activity of lectin’s partition coefficient (*K*) and the purification factor of lectins in the top phase (*PF*) (detailed calculation method is in Section 3.7), the ATPS rendering the most-effective partitioning was chosen for further study.

#### 2.1.1. Effect of Concentration of (NH_4_)_2_SO_4_

**Figure 1:**
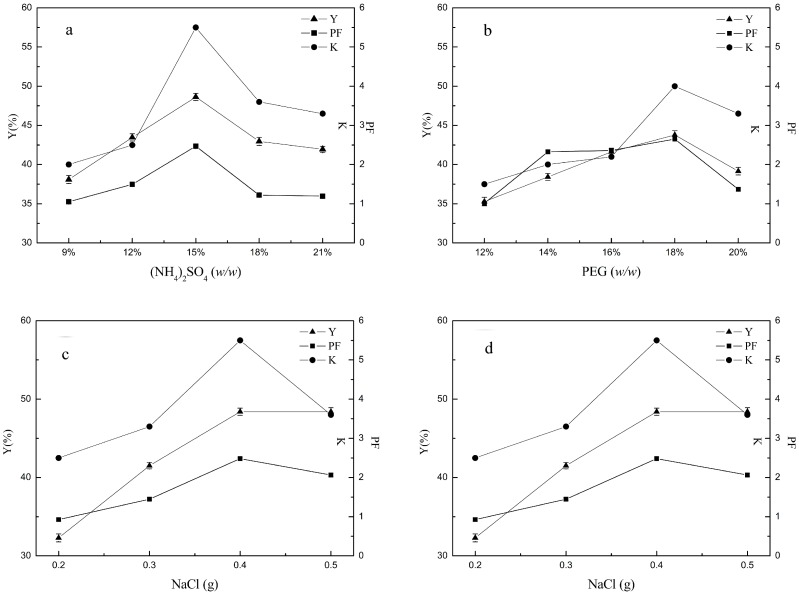
Comparison of single factor results. (**a**) Effect of concentration of (NH_4_)_2_SO_4_ on the extraction; (**b**) effect of PEG on the extraction; (**c**) effect of NaCl on the extraction; (**d**) effect of pH on the extraction (where *Y*: the protein recovery; *PF*: the purification factor of lectin; *K*: lectin partition coefficient).

As indicated in Figure 1a, *PF* increased with the concentration of (NH_4_)_2_SO_4_ until the mass fraction reached 15%. The trend of *Y* and *K* changed in a similar way. When the mass fraction of (NH_4_)_2_SO_4_ ranged from 9% to 15%, the ionic strength of the salt-rich bottom phase increased with the increase of (NH_4_)_2_SO_4_ concentration. The presence of high concentrations of salts increased the hydrophobicity of the bottom phase [22], in which the stability of the protein in the solution was broken. Because of the competition between the large number of salt ions and proteins for water molecules, the solvation spheres surrounding the proteins’ ionized groups were removed [23]. This could be due to the ‘‘salting-out effect’’. According to Babu [24], the solubility of biomolecules decreased with an increase in the salt concentration in the bottom phase, which led to the increased partitioning of biomolecules to the top phase. At mass fraction above 18%, *PF*, *K* and *Y* in the top phase were gradually decreased. One possible reason was the denaturation of lectins caused by the salting-out effect. Another reason was that the lectins precipitated at the interphase were discarded.

#### 2.1.2. Effect of Concentration of PEG

To study the effect of concentration of PEG 600, different masses of PEG 600 were mixed with (NH_4_)_2_SO_4_ to form ATPS. Partitioning was performed as described previously. As indicated in Figure 1b, the trends of *PF*, *K* and *Y* in the PEG 600/(NH_4_)_2_SO_4_ ATPS were similar to those in Section 2.1.1, showing a tendency to increase first and then decrease with the increase of the mass fraction of PEG 600. Overall, the increasing degree of *PF* was larger than that of *Y*, which meant lectins were more preferably distributed to the top phase than residual proteins under that condition. The reason for the difference between the distribution of lectins and residual proteins is attributed to properties of the proteins, such as the molecular weight, shape, volume and surface area [25]. Electrostatic interactions and the salting-out effect were driving forces of the protein distribution in the ATPS, but the main driving force was the hydrophobic interaction [26]. Tubio [27] considered that for PEG of low molar mass (600–3350 g/mol), the strong interaction between PEG and protein accelerated the protein to transfer to the top phase. The more-hydrophobic tetramer form probably interacted preferentially with the more-hydrophobic PEG-rich phase, enhancing the extraction of lectins to the top phase [19]. Generally, the partition coefficient of proteins became more polarized as the polymer concentration increased, or increased up to a certain value and then decreased [28]. In this case, *PF*, *K* and *Y* decreased after the mass fraction of PEG 600 reached 18%. An increase in PEG concentration increased the excluded volume of PEG, which in turn reduced the solvent volume fraction. Protein was excluded by the phase-forming polymer.

#### 2.1.3. Effect of NaCl on the Extraction

NaCl addition has been used to enhance selectivity for many ATPS [17,29,30]. In this case, as shown in Figure 1c, an improvement in *PF*, *K* and *Y* of the lectins were observed as the concentration of NaCl increased. *PF*, *K* and *Y* in the PEG 600/(NH_4_)_2_SO_4_ ATPS increased with the increase of the quantity of NaCl added. It has been shown that ions of added salts are distributed unequally between the phases, leading to an electrostatic potential difference between the phases [30]. This raised the possibility of changing the partition coefficient of specific proteins according to their charge. When the quality of NaCl was more than 0.4 g, *PF* decreased while *Y* remained around 50%, which indicated that lectins were salted out of the top phase. In terms of purity, the best partitioning was achieved in the ATPS contained 0.4 g NaCl.

#### 2.1.4. Effect of pH

The lectin of *Phaseolus vulgaris* seeds was structurally active in a state of dimer–tetramer equilibrium in the range of pH between 5.0 and 7.0 or more, with the content of different polymer forms increasing [31,32,33].

The pH affected the charge, the ion composition, the surface character of the targeted protein and the variation in the partitioning into the top and bottom phases [34]. The variation of *PF*, *K* and *Y* with pH over the range 6.5–8.5 is shown in Figure 1d. With the increase of pH value, *PF*, *K* and *Y* increased first and then decreased, reaching the maximum at pH 7.5, indicating a strong dependence of pH on partition behavior. As a general rule in PEG–salt systems, most proteins with acidic isoelectric points (pI), and consequently negative surface charges, prefer the top phase, while positively charged proteins move to the bottom phase [35]. As shown in Figure 1d, when pH was raised from 6.5 to 7.5, higher than the pI of lectins [32] (pI value between 5.2 and 6.0), the variations of *PF*, *K* and *Y* were increased. When pH was higher than 7.5, with the content of tetrameric form increasing with the pH [36], a decrease in the variation of *PF*, *K* and *Y* of lectins was observed due to size-exclusion effects [19]. Thus, the optimal values for the partition parameters were obtained at pH 7.5.

From the above analysis, it could be concluded that the best single-factor condition was as follows: ATPS was formed with ammonium sulfate 15% (*w*/*w*), PEG 18% (*w*/*w*), NaCl 0.4 g, adding 1 mL crude extract solutions. The total weight of the system was 5 g with pH 7.5.

### 2.2. Response Surface Analysis

#### 2.2.1. Statistical Analysis and Model Fitting

The data were processed by Design Expert (Version 8.0.6) data-processing software. Box–Behnken design (BBD) with four independent factors set at three variation levels was carried out (Table 1). The second-order prediction models with response value (*Y*_1_) and response value (*Y*_2_) were obtained. The fitting equations were:

*Y*_1_ = 41.04 + 1.52*x*_1_ + 1.61*x*_2_ + 2.08*x*_3_ − 0.24*x*_4_ − 1.62*x*_1_*x*_2_ − 0.45*x*_1_*x*_3_ − 0.17*x*_1_*x*_4_ − 0.98*x*_2_*x*_3_ + 1.82*x*_2_*x*_4_ − 0.23*x*_3_*x*_4_ − 4.91*x*_1_^2^ − 5.34*x*_2_^2^ − 4.46*x*_3_^2^ − 4.99*x*_4_^2^ (1)

*Y*_2_ = 3.17 − 0.064*x*_1_ − 0.12*x*_2_ + 0.098*x*_3_ + 0.033*x*_4_ + 0.17*x*_1_*x*_2_ − 0.24*x*_1_*x*_3_ − 0.23*x*_1_*x*_4_ − 0.15*x*_2_*x*_3_ − 0.18*x*_2_*x*_4_ + 0.27*x*_3_*x*_4_ − 0.70*x*_1_^2^ − 0.62*x*_2_^2^ − 0.68*x*_3_^2^ − 0.52*x*_4_^2^ (2)

#### 2.2.2. Analysis of Variance

Analysis of variance (ANOVA) for the models *Y*_1_ and *Y*_2_ is shown in Table 2 and Table 3. The regression models were highly significant (*p*_1_ < 0.01, *p*_2_ < 0.01), while the lack-of-fit tests were not significant (*p*_1_ = 0.1771 > 0.05, *p*_2_ = 0.2737 > 0.05). The determination coefficients (R_1_^2^ and R_2_^2^) of the predicted models were 0.9652 and 0.9873, indicating that only 3.48% and 1.27% of the total variation were not explained by the models. Meanwhile, a very low value of 3.6% and 4.06% for the coefficient of variation (CV) clearly indicated that the experimental values were associated with a very high degree of precision and a good deal of reliability. Thus, the models explained the response adequately.

#### 2.2.3. Interactive Analysis

Three-dimensional (3D) response-graph analysis provided an intuitive means of analyzing the effect of the interaction between the trends of the variables.

**Figure 2:**
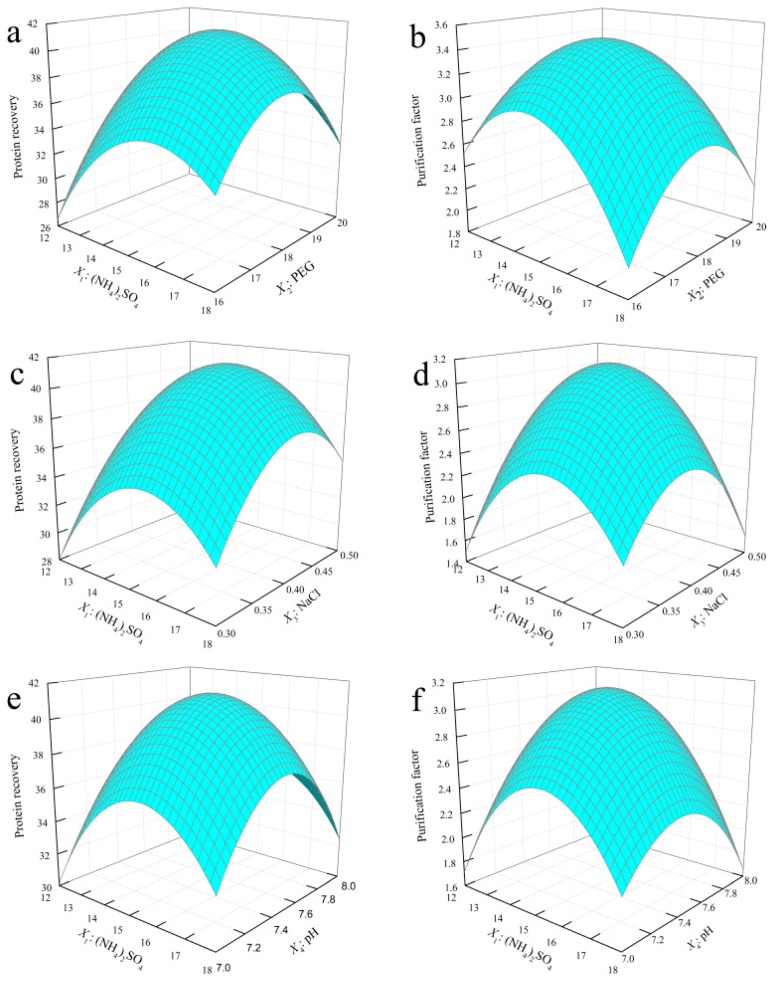
Interactive analysis. Three-dimensional (3D) response surface curves for the effects of (NH_4_)_2_SO_4_ (*X*_1_), PEG (*X*_2_), NaCl (*X*_3_) and pH (*X*_4_) in protein recovery (**a**,**c**,**e**) and purification factor (**b**,**d**,**f**).

Figure 2a,b shows that, at the highest level (+1) of the PEG of the aqueous phase, *PF* and *Y* increased to the highest level with increase of the mass fraction of (NH_4_)_2_SO_4_; however, as the mass fraction of (NH_4_)_2_SO_4_ continued to increase, both *PF* and *Y* began to decline. At the lowest level (−1) of the PEG, *PF* and *Y* increased first and then decreased with the increase of the mass fraction of (NH_4_)_2_SO_4_. The reason was that when the PEG value and (NH_4_)_2_SO_4_ value were both at the −1 level, hydrophobic interactions played probably the major role in the fractionation of proteins in ATPS, which is involved in two such interactions: the phase hydrophobicity effect and the salting-out effect [37]. The lower ionic strength and weak hydrophobic interactions were not conducive to the movement of lectins to the top phase. With the increase of PEG and (NH_4_)_2_SO_4_, the phase hydrophobicity effect was directly related to the chemical identity of the constituents of the ATPS, as well as their concentration [38]. As ionic strength and hydrophobic interaction increased gradually, lectins were more easily distributed into top phase, causing *PF* and *Y* to increase. However, as the two values continued to increase, the volume of the top phase decreased to a certain value and ionic strength increased in the bottom phase, resulting in the protein to be excluded from the two phases.

Figure 2c,d shows that the effect of the interaction between the quantity of NaCl and the mass fraction of (NH_4_)_2_SO_4_ on *PF* and *Y* was similar to that of the mass fraction of (NH_4_)_2_SO_4_ and PEG, because of the effect on hydrophobic interactions and salting-out in ATPS. The Hofmeister or lyotropic series describes the salting-out effects of ions on proteins [39]. The hydrophobicity of the anions studied was in the following order: ClO_4_^−^ > Cl^−^ > CH_2_COO^−^ > HPO_4_^2^^−^/H_2_PO^4^^−^, showing that the partitioning of the salt to the relatively more-hydrophobic top phase was enhanced with increases in the hydrophobicity of the anion [40]. In addition, specific interactions between salts and proteins are believed to be responsible for proteins’ partitioning efficiency [41]. The type and concentration of salts had a significant effect on protein partition and extraction efficiencies in a PEG–salt ATPS. The influence of salt on the partitioning of proteins was caused by the non-uniform distribution of salt ions in the two phases, by the electric current difference and by the salt distribution inequality [42]. The ATPS might have become positively charged in the top phase and negatively charged in the bottom phase, which improved the protein movement to the other phase by electrostatic repulsion effects. In this work, the lectin was negatively charged at the pH studied (7.5, 8.0 and 8.5), due to a pI value between 5.2 and 6.0 [32], favoring its movement to the top phase [42].

All of the above conditions contributed to enhancing the distribution of lectins in top phase. However, further addition of NaCl and (NH_4_)_2_SO_4_ would lead to salt precipitation in the ATPS, because the solution had reached its saturation point and can no longer dissolve any more NaCl and (NH_4_)_2_SO_4_.

It can be seen from Figure 2e,f that the trends of *PF* and *Y* were similar to those in Figure 2a–d. When pH was higher than 7.0 (higher than the isoelectric point of lectin), the protein had an overall negative charge and repulsions were expected to occur between the protein and the negatively charged sulfate radical anion [19]. The balance of these repulsion effects yielded the optimum pH of 7.5. In addition, the more-hydrophobic tetramer form probably interacts preferentially with the more-hydrophobic PEG-rich phase, enhancing the extraction of lectin to the polymer-rich phase and explaining the increase of *PF* and *Y* with the increase of pH.

#### 2.2.4. Validation of the Best Extraction Conditions

The optimal extraction conditions were as follows: the mass fraction of (NH_4_)_2_SO_4_ was 15.38%, the mass fraction of PEG was 18.22%, the quantity of NaCl was 0.42 g, the crude extract volume was 1 mL and the pH of the system was 7.49. Under these conditions, the predicted protein recovery was 41.45% and the predicted lectin purification factor was 3.16. In order to facilitate the operation, the predicted optimal process conditions were amended as follows: the mass fraction of (NH_4_)_2_SO_4_ was 15%, the mass fraction of PEG 600 was 18%, the quantity of NaCl was 0.4 g, the crude extract volume was 1 mL and the pH of the system was 7.5. After three experiments, the protein recovery was 42.32 ± 0.98% and the lectin purification factor was 3.08 ± 0.07, which was not different from the predicted value and indicated that the model was better.

### 2.3. Electrophoretic Analysis

**Figure 3:**
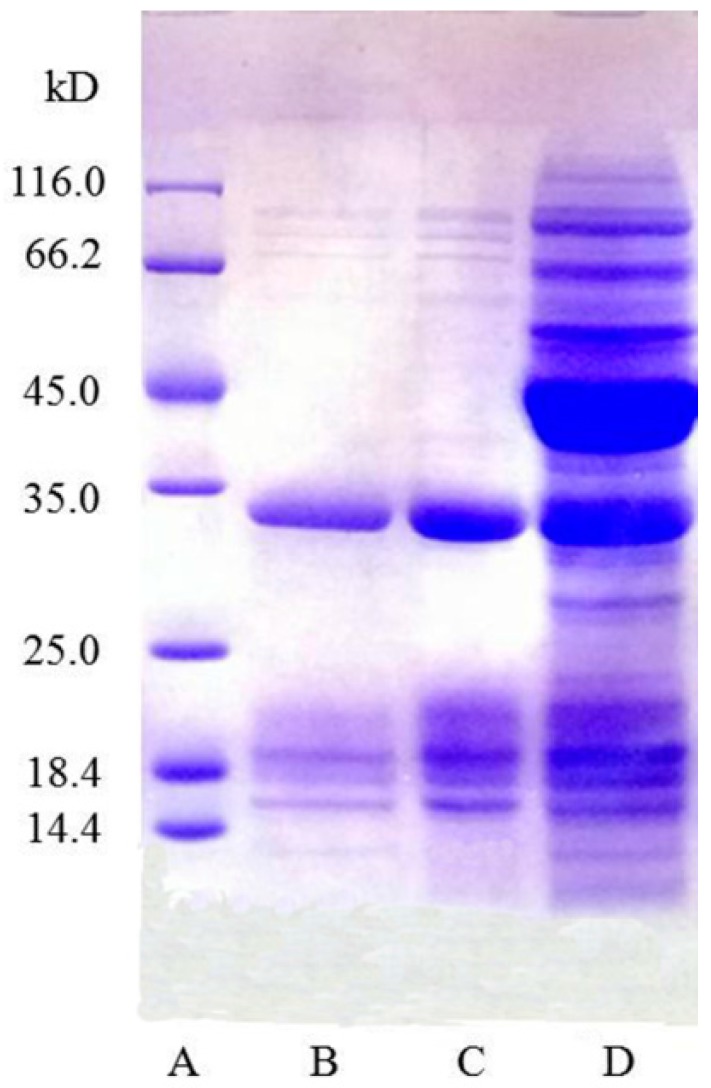
SDS–PAGE of lectin. Lane A, molecular mass standards (in kDa); lane B, sample (5 μg) purified by ATPS with PEG/ammonium sulfate; lane C, sample (10 μg) purified by ATPS with PEG/ammonium sulfate; lane D, crude extract of Zihua snap-bean (*Phaseolus vulgaris)* seeds (10 μg).

Figure 3 shows the SDS–PAGE (sodium dodecyl sulfate polyacrylamide gel electropheresis) of the lectin (from the Zihua snap-bean seeds after the ATPS process) found in the top phase (lanes B,C), and from the crude extract of the Zihua snap-bean seeds (lane D). The electrophoretic analysis of the crude extract showed multiple bands (lane D), while the SDS–PAGE of the lectin after the ATPS showed three bands (lanes B,C) with a major band at 35 kDa and two other bands at 18.4 and 14.4 kDa. The major band (35 kDa) was similar to that obtained by He [16]. The purity of the lectin was increased by the ATPS process.

### 2.4. Comparison of Different Methods

**Figure 4:**
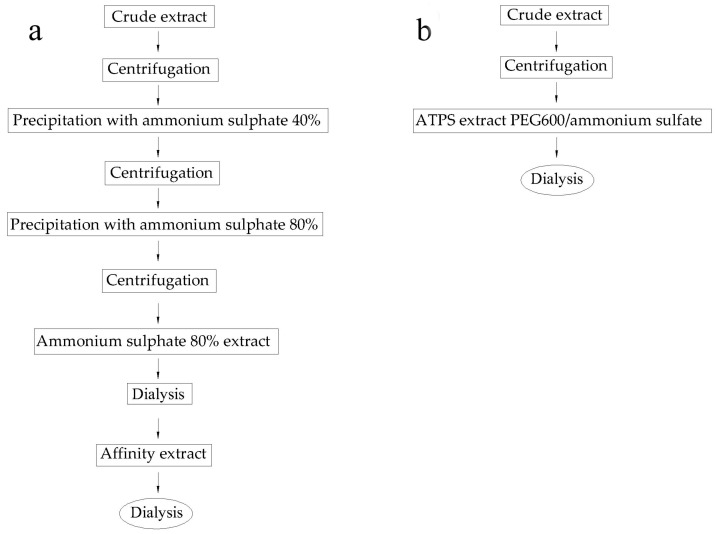
Purification of lectin using affinity chromatography (**a**) and an aqueous two-phase system (**b**).

The affinity chromatography process for kidney-bean lectin purification [43] has been used in biological applications. This purification process includes ten operational steps (Figure 4a), compared with the ATPS process which includes four operational steps (Figure 4b). When the crude extract from the Zihua snap-bean (*Phaseolus vulgaris*) seeds was applied to the PEG 600/ammonium sulfate system, an overall reduction in the number of purification steps was therefore realized (from 10 to 4). In addition, a higher yield (42.32%) was obtained by the ATPS method when compared with the average yield (about 35%) of the common plant-lectin-extraction methods [10], and the activity of lectin after purification was similar to that of affinity chromatography [11]. However, the yield and *PF* are related not only with the method of purification but also with the type of starting material [10]. For the industrial production and extensive commercialization of lectin, a large-scale process should be designed to minimize the number of steps in the purification, while maintaining high yields and product purity, quality and activity. Common lectin purification methods include precipitation with salts [9], organic solvents [15] and various chromatographic techniques [11], all of them showing difficulties in large-scale applications [44]. 50% to 90% of the production costs of biological products are determined by the purification strategy [10]. Thus, the simplicity of ATPS processes, combined with a low cost and easy scale-up, makes this unit operation attractive for large-scale lectin processing.

## 3. Materials and Methods

### 3.1. Instruments

Solution pH was determined by a FE201EL20 pH meter, and an AL-04 electronic analytical balance (Mettler Toledo Instruments Co., Ltd., Shanghai, China) was used to measure the weight of sample. CT14D desktop high-speed centrifuge (Shanghai Techcomp Scientific Instrument Co., Ltd., Shanghai, China), and an A150011-Type vortex mixer (Nanjing Jiajun Biological Co., Ltd., Nanjing, China) were used for the sample treatment. The T6 Xinshiji ultraviolet visible spectrophotometer was from Beijing Purkinje General Instrument Co., Ltd. (Beijing, China) The Biorad Mini-PROTEAN Tetra Cells 4-Gel 165-8004 was from Bio-Rad Co., Ltd. (Guangzhou China).

### 3.2. Reagents

Polyethylene glycol (PEG) with average molecular weight (MW) of 600 Da was from Sinopharm Chemical Reagent Co., Ltd. (Shanghai, China). Ammonium sulfate ((NH_4_)_2_SO_4_), disodium hydrogen phosphate (Na_2_HPO_4_), monometallic sodium orthophosphate (NaH_2_PO_4_) and sodium chloride (NaCl) were obtained from Beijing Yili Fine Chemicals Co., Ltd. (Beijing, China). 2% rabbit red-blood-cell suspension was from Beijing Baiaolaibo Technology Co., Ltd. (Beijing, China). SDS–PAGE electrophoresis kit and Folin’s phenol reagent were from Beijing Solarbio Technology Co., Ltd. (Beijing, China). Ultrapure water was obtained from Northeast Agricultural University. All of the reagents were used without further purification.

### 3.3. Preparation of Extract

Zihua snap-bean (*Phaseolus vulgaris*) seeds were purchased from Harbin Xiangfang District Gongbin Seed Company (Harbin, China). For the extraction of lectin, dried Zihua snap-bean seeds were ground to a fine powder which was passed through a 50-mesh sieve. Then, the powder was mixed with 10 volumes of phosphate-buffered saline (PBS, 10 mM, pH 7.0) by agitation overnight at 4 °C. Afterwards, the extract was filtered through the filtrate, centrifuged at 10,000× *g* for 30 min, and the supernatant was used as crude extract for further extraction.

### 3.4. Preparation of ATPS

The ATPS was formed by mixing the appropriate weight of the stock solutions of ammonium sulfate, PEG 600 and NaCl in 10 mL graduated centrifuge tubes. 1 mL of crude extract solution (adjusting the crude extract concentration to 15 mg/mL) was added to the ATPS. Ultrapure water was used to bring the final weight of the system to 5 g. The pH levels of the systems were adjusted to the desired condition using NaOH (1 mol/L). The compounds were mixed by a vortex mixer. The complete phase separation was achieved by centrifuging at 2000× *g* for 10 min at 25 °C. After phase volumes were measured, the top phases were collected to measure protein concentration and the hemagglutinating activity, and the bottom phases were collected to measure the hemagglutinating activity.

### 3.5. Protein Determination

The protein content of the top phase of the systems was determined spectrophotometrically using Folin’s phenol method [45]. To avoid interference from the phase-formed substance in the top phase, all samples were analyzed against blanks containing the same phase composition without proteins. Using BSA (Albumin from bovine serum) as standard, the samples were read at 650 nm.

### 3.6. Determination of the Hemagglutinating Activity

The determination of the hemagglutinating activity (HA) was performed in microtiter plates according to Correia and Coelho [46].

The lectin preparation (50 μL) was twofold serially diluted with stroke-physiological saline solution, and 50 μL of 2% rabbit red-blood-cell suspension was added. The results were read after 45 min at room temperature, when the negative control had fully sedimented. The hemagglutination titer (HU) was defined as the reciprocal of the maximal dilution of the sample that gave visible agglutination. The hemagglutination activity (HU/mg) was expressed as the amount of hemagglutination titer per mg protein.

### 3.7. Definition of Parameters in ATP Systems

The separation performance of extracting lectin from the seed by ATPS was evaluated by determining several parameters.

The hemagglutinating activity of the lectin partition coefficient (*K*) was defined as the ratio of the maximum dilution factor of the hemagglutination test in the top phase (*N*_T_) to that in the bottom phase (*N*_B_):

(3) $$K=\frac{N_{T}}{N_{B}}.$$

The protein recovery in the top phase (*Y*) was defined as the ratio between the mass of protein in the top phase and the total mass of protein added to the system, and expressed in percentage:

(4) $$Y=\frac{\mathrm{Total}\;\mathrm{mass}\;\mathrm{of}\;\mathrm{the}\;\mathrm{top}\;\mathrm{phase}\;\mathrm{protein}}{\mathrm{Total}\;\mathrm{mass}\;\mathrm{of}\;\mathrm{protein}\;\mathrm{added}\;\mathrm{to}\;\mathrm{the}\;\mathrm{system}}\times 100\%.$$

The purification factor of lectin in the top phase (*PF*) was defined as the ratio of the hemagglutinating activity of lectin in the top phase to that in the crude extract:

(5) $$PF=\frac{\mathrm{the}\;\mathrm{hemaggluti}\;\mathrm{nating}\;\mathrm{activity}\;\mathrm{of}\;\mathrm{lectins}\;\mathrm{in}\;\mathrm{the}\;\mathrm{top}\;\mathrm{phase}}{\mathrm{the}\;\mathrm{hemaggluti}\;\mathrm{nating}\;\mathrm{activity}\;\mathrm{of}\;\mathrm{lectins}\;\mathrm{in}\;\mathrm{the}\;\mathrm{crude}\;\mathrm{extract}}.$$

### 3.8. Experimental Design

In order to optimize the interaction between the extraction conditions and parallel factors, the experimental results were carried out on the basis of a single-factor experiment. The experiment was carried out by using the response surface method (RSM) experiment. The effects of various factors, including *X*_1_ (the mass fraction of (NH_4_)_2_SO_4_), *X*_2_ (the mass fraction of PEG), *X*_3_ (the quantity of NaCl) and *X*_4_ (pH value), were studied, and the experimental design is shown in Table 4. The protein recovery (*Y*) and the purification factor of lectin (*PF*) in the top phase were taken as the responses of the experiments, and the optimum process parameters were optimized. Each experiment was replicated three times.

A second-order polynomial equation model was used to fit the *Y* and *PF* data with interaction terms as given below:

*Y_i_ = A*_0_*+ A_i_Σx_i_ + A_ij_Σx_i_x_j_ + A_ii_Σx_i_^2^ (i≠j),*

where *Y_i_* is the predicted response value; *A*_0_*, A_i_, A_ij_* and *A_ii_* are the regression coefficients of the model for the intercept, linear, cross-product and quadratic terms, respectively; and *x_i_* is the variable studied [47,48].

### 3.9. Electrophoresis

SDS–PAGE was carried out in order to evaluate the purity of protein in the phases, using 12% bis-acrylamide homogeneous gel. The gel was run for approximately 95 min using tris-glycine–SDS running buffer at constant voltages of 80 V for stacking gel and 120 V for separating gel. After the end of the electrophoresis, the tape was washed with distilled water for several times, stained with Coomassie brilliant blue R-250 for 30 min and decolorized with eluent.

## 4. Conclusions

The optimum ATPS conditions for separation of lectin from Zihua snap-bean (*Phaseolus vulgaris*) seeds were obtained using response surface methodology and a Box–Behnken experimental design. The effects of processing parameters, such as *X*_1_ (the mass fraction of (NH_4_)_2_SO_4_), *X*_2_ (the mass fraction of PEG 600), *X*_3_ (the quantity of NaCl) and *X*_4_ (the pH value), were successfully evaluated by the regression models and the three-dimensional (3D) response surface curves. The results showed that the optimal conditions for the separation of lectin from Zihua snap-bean seeds with ATPS were as follows: 15% (NH_4_)_2_SO_4_ (*w*/*w*), 18% PEG 600 (*w*/*w*), 0.4 g/5 g NaCl, and 1 mL of added Zihua snap-bean seed crude extract, while the pH of the system was 7.5. Under the optimal conditions, most of the lectin was assigned to the top phase in ATPS, and the hemagglutination activity of the purified top phase was 3.08 times greater than that of the crude extract. The ATPS extraction process was proved to be efficient based on the purity determination of lectin using SDS–PAGE analysis. Therefore, it was suggested that the ATPS could be a valuable protocol for use in the laboratory and in industrial processes for the purification of lectin from Zihua snap-bean seeds.

## Figures and Tables

**Table 1:** BBD and the results (means of triplicate tests) for the protein recovery and purification factor of lectin.

Number	*X*_1_ (NH_4_)_2_SO_4_ (*w*/*w*) %	*X*_2_ PEG 600 (*w*/*w*) %	*X*_3_ NaCl g	*X*_4_ pH	Protein Recovery (%)	Purification Factor (Fold)
1	0	1	0	−1	32.5	1.85
2	1	0	0	−1	35.0	1.71
3	0	0	−1	1	40.9	3.18
4	1	−1	0	0	30.2	1.98
5	0	0	0	0	25.7	2.24
6	0	0	0	0	30.5	1.80
7	0	1	0	1	32.0	1.72
8	0	−1	0	−1	32.0	1.72
9	−1	1	0	0	33.0	1.82
10	0	0	0	0	41.6	3.12
11	0	0	1	1	33.8	2.37
12	1	1	0	0	33.1	2.26
13	0	0	−1	−1	34.8	1.58
14	1	0	−1	0	32.3	1.86
15	0	−1	−1	0	39.7	3.28
16	0	1	1	0	25.9	1.84
17	1	0	0	1	31.8	1.73
18	0	−1	0	1	31.7	1.90
19	−1	0	−1	0	33.2	1.66
20	0	0	0	0	32.3	2.09
21	−1	0	1	0	29.3	2.13
22	0	1	−1	0	41.5	3.13
23	0	0	0	0	31.5	1.59
24	1	0	1	0	29.5	2.37
25	0	0	1	−1	26.8	2.33
26	−1	−1	0	0	31.2	1.84
27	0	−1	1	0	33.5	2.09
28	−1	0	0	1	41.5	3.13
29	−1	0	0	−1	26.9	1.39

**Table 2:** The variance analysis of the fitted quadratic polynomial prediction model of *Y*_1._

Source	Sum of Squares	df	Mean Square	F	*p*_1_-Value
Model	545.13	14	38.94	27.72	<0.0001
Residual	19.67	14	1.4		
Lack of fit	17.11	10	1.71	2.68	0.1771
Pure error	2.55	4	0.64		
Cor total	564.79	28			
CV%			3.6%		
R_1_^2^			0.9652		

**Table 3:** The variance analysis of the fitted quadratic polynomial prediction model of *Y*_2__._

Source	Sum of Squares	df	Mean Square	F	*p*_2_-Value
Model	8.12	14	0.58	77.69	<0.0001
Residual	0.1	14	7.47 × 10^−3^		
Lack of fit	0.087	10	8.67 × 10^−3^	1.94	0.2737
Pure error	0.018	4	4.47 × 10^−3^		
Cor total	8.23	28	0.58		
CV%			4.06%		
R_2_^2^			0.9873		

**Table 4:** Factors and levels in the response surface design used for optimization of separated lectin by ATPS.

Variables	Coded Variable Levels		
	−1	0	+1
*X*_1_ (NH_4_)_2_SO_4_ (*w*/*w*)%	12	15	18
*X*_2_ PEG 600 (*w*/*w*)%	14	16	18
*X*_3_ NaCI/g	0.3	0.4	0.5
*X*_4_ pH	7.0	7.5	8.0
